# The Development of Floral Scent Research: A Comprehensive Bibliometric Analysis (1987–2022)

**DOI:** 10.3390/plants12233947

**Published:** 2023-11-23

**Authors:** Qin Peng, Yangyang Zhang, Junjun Fan, Anil Shrestha, Wangxiang Zhang, Guangyu Wang

**Affiliations:** 1 College of Forestry, Nanjing Forestry University, No. 159 Longpan Road, Xuanwu District, Nanjing 210037, China; penqin@njfu.edu.cn; 2 Department of Forest Resources Management, Faculty of Forestry, The University of British Columbia, 2424 Main Mall, Vancouver, BC V6T 1Z4, Canada; yangyangzhang1993@outlook.com (Y.Z.); anil.shrestha@ubc.ca (A.S.); 3 College of Horticulture and Forestry Sciences, Huazhong Agricultural University, No. 1 Shizishan Street, Hongshan District, Wuhan 430070, China; 4 College of Horticulture, Jinling Institute of Technology, No. 99 Hongjing Avenue, Jiangning District, Nanjing 211169, China; joycefan2019@jit.edu.can

**Keywords:** floral scent, volatile compounds, pollination, systematic review

## Abstract

Floral scent (FS) plays a pivotal role in maintaining ecological equilibrium within plant populations and ecosystems while also bearing significance for human well-being. Despite the growing interest in FS research, there exists a dearth of comprehensive analyses on research trends, contemporary topics, and their broader implications. In this study, we employ bibliometric techniques using data from the Web of Science Core Collection spanning 1987–2022 to offer a quantitative overview of the scientific literature surrounding FS by examining the annual publication outputs, popular research areas, temporal trends in keywords, geographic distribution of relevant studies, institutions, co-organizations, as well as relevant authors. Our findings reveal a marked upsurge in FS publications, notably within the domains of Food Science Technology, Plant Sciences, Chemistry, Agriculture, Biochemistry, and Molecular Biology. The research landscape in FS primarily encompasses evolutionary dynamics, volatile compound analyses, biosynthesis mechanisms, and essential oil properties. These research trends signify a transition from micro-level exploration, focusing on individual pollination ecological functions of FS, to a macro-perspective that emphasizes FS’s overarching impact on species diversity and ecosystem stability. This shift extends from the investigation of singular sensory attributes of FS to a holistic evaluation of their role in food production, quality, and yield enhancement. It encompasses a move away from mere FS extraction towards the examination of antioxidant potential within phenolic compounds and other industrial applications. Thus, improving research methodologies, strengthening interdisciplinary collaboration on an international scale, and delving deeper into the multifaceted ecological functions of floral diversity and their societal implications will be paramount.

## 1. Introduction

Floral scents (FSs), composed of a spectrum of low molecular weight (30–300 amu) volatile compounds [1,2], have become a subject of intense interdisciplinary interest, attracting researchers from botany, chemistry, ecology, and cultural studies. This collective fascination has yielded invaluable insights into the chemical constituents underpinning FSs, their multifaceted ecological functions, and their intriguing impact on human emotions and behaviors. Indeed, FSs represent a pivotal functional trait shaping interactions between flowers and insects in ecosystems [3]. These pollination systems can exhibit specialization [4,5] or generality [6,7], and the release of FSs is intricately regulated by a host of biotic or abiotic factors [8], ranging from drought stress [9] to temperature [10], profoundly influencing a flower’s allure to pollinators. Notably, the ramifications of habitat loss affect plant–pollinator dynamics, with direct consequences for plant reproduction [11] and thus relate to the functional areas of ecosystems [12]. The evolution of practical methodologies and affordable instrumentation for collecting, separating, and identifying volatile compounds [13,14,15] has solidified FS research as a cornerstone in scientific inquiry. These aromatic compounds have now been identified in nearly 1000 plant species [16], with their applications spanning diverse sectors, from pharmaceuticals [17] to cosmetics [18,19] to the culinary realm [20], with key components like benzaldehyde, benzyl alcohol, acetophenone, phenylacetaldehyde, and methyl benzoate enhancing the sensory experience [21,22]. FSs extend their influence on human well-being, nurturing physical, mental, and emotional health through direct contact with plant materials or the use of essential oils [23,24]. Their contributions are not confined to individual well-being; they play an indispensable role in activities such as forest therapy [25] and multi-sensory interactions within urban forests and national parks [26,27]. Despite the wealth of research conducted since 1987, encompassing chemical analyses, ecological roles, pollinator dynamics, associations with climate change, habitat destruction, and other factors [28], an econometric perspective to comprehensively understand the evolution of FS research trends, as evidenced by VOSviewer data [29], remains untapped.

In our study, we employ social network analysis to quantitatively assess these patterns and shifts in FS research [28,29] with the following research questions:

1. How have the interdisciplinary interests and the advancement in technology influenced the study of FS?
2. How do high-frequency keywords, extracted from bibliometric data, reflect the areas of emphasis in FS research, and what can these keywords reveal about the dominant themes and connections within this scientific domain?
3. What are the key trends and thematic shifts identified through bibliometric analysis in the FS field, and how can these patterns inform interdisciplinary collaboration and future research directions?

Our aim is to unearth research gaps, accentuate focal areas, and foster interdisciplinary cooperation, ultimately enriching our appreciation of the ecological, biological, cultural, and industrial significance of FS, thereby shedding light on the intricacies of plant–environment interactions and their far-reaching implications.

## 2. Methods

### 2.1. Data Collection

To fulfill this objective, we selected the Web of Science database due to its exceptional bibliometric analysis capabilities, offering an extensive coverage period and a user-friendly interface when compared to Scopus [30]. This bibliometric collection of the global scientific literature on floral scents was assembled in February 2023, utilizing the following Web of Science Core Collection (WoSCC) citation indexes:

1. Science Citation Index Expanded (SCIE) 1900–present;
2. Social Sciences Citation Index (SSCI) 1956–present;
3. Arts & Humanities Citation Index (A&HCI) 1975–present;
4. Emerging Sources Citation Index (ESCI) 2018–present;
5. Conference Proceedings Citation Index-Science (CPCI-S) 1990–present;
6. Conference Proceedings Citation Index-Social Sciences and Humanities (CPCI-SSH) 1990–present.

We employed the following keywords (“flower* aroma*,” “flower* scent*,” “flower* fragrance*,” “flower* volatiles*,” “floral* aroma*,” “floral* scent*,” “floral* fragrance*,” “floral* volatiles*”) to search document titles, abstracts, and keywords on Web of Science (https://www.webofscience.com/wos/woscc/summary/65b2c9b4-a66d-40cf-b290-e6981a2d9d30-7213ff82/relevance/1, accessed on 5 April 2023). Our study period began with the earliest publication in 1965 and given that only two studies were published annually before 1987, we focused on the period from 1 January 1987 to 31 December 2022. This selection process yielded a total of 5049 distinct articles.

### 2.2. Data Analysis

We used HistCite (Pro 2.1 version) and VOSviewer (1.6.19 version) for data visualization, figure generation, and various data-mining tasks, including the extraction of annual output counts, documentation of article types, journal sources, contributing institutions, and author profiles. HistCite (Pro2.1), a software tool developed by Garfield and colleagues [31,32], serves as an invaluable resource for exploring the dynamics within knowledge domains. It accomplishes this by processing bibliographic records, including referenced citations, to yield information metrics tables. The software’s versatility spans a wide range of applications [33,34,35], and in our case, we employed it to scrutinize research trends in the field of floral scents (FSs). HistCite aided in computing the Average Local Citation Score (ALCS) and Total Local Citation Score (TLCS), shedding light on how frequently papers within the FS database were cited in other scholarly articles. 

VOSviewer (1.6.19), a robust and freely available software tool for bibliometric analysis and visualization [36], played a pivotal role in generating network graphs. These visualizations are rooted in the degree of association between items displayed, facilitated by co-occurrence network clustering and density analysis. VOSviewer allowed us to construct a co-occurrence map highlighting thematic trends in FS research. Specifically, the “keywords in co-occurrence” network unveiled the connections between pairs of keywords by ascertaining the number of publications where they coexist in the title, abstract, or keywords list. To accentuate the most significant keywords visually, we confined the analysis to “title” and “abstract” sections and applied filters, such as “Ignore structured abstract labels” and “Ignore copyright statements.” To ensure precision and eliminate redundancy, we incorporated a synonym file to unify keywords pertaining to the same topic before reimporting for processing.

VOSviewer’s overlay visualization feature allowed us to observe network items on a temporal gradient, providing insight into the chronological co-occurrence of these items. This visualization was constructed by assessing the average publication year in which keywords made their appearance [37]. In an effort to refine the visual representation in VOSviewer, we set a threshold of 50 occurrences for keywords present in article titles and abstracts, ultimately yielding 68 high-frequency keywords after eliminating invalid terms.

## 3. Results

### 3.1. Temporal Evolution of Research Outputs

**Figure 1:**
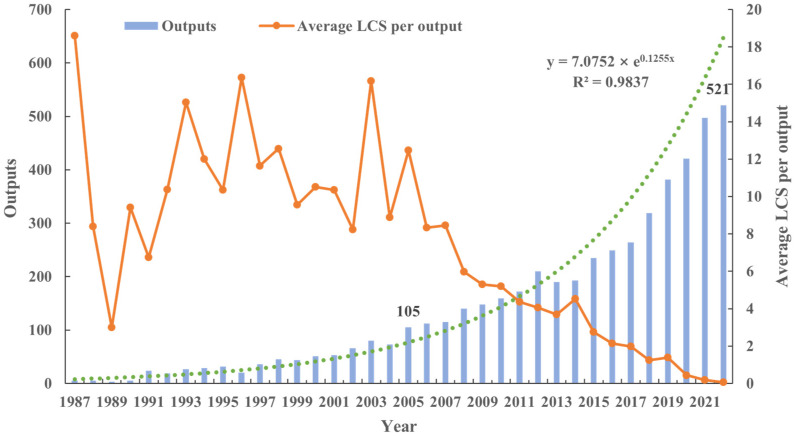
Temporal evolution of outputs on FS research. The term “LCS” (local citation score) denotes the frequency at which papers authored by an individual in the FS database are referenced by other research papers. Note: The green dotted line represents exponential trendline of outputs on FS research.

The temporal evolution of research outputs is illustrated in Figure 1, spanning the period from 1987 to 2022. The publication count exhibited a remarkable progression, starting with a mere 5 publications in 1987 and surging to 521 in 2022, representing a remarkable 100-fold increase. The growth trajectory remained relatively steady between 1987 and 2005, after which it gained significant momentum. Conversely, the average number of citations per publication displayed an inverse relationship to the surge in publications, as depicted in Figure 1. A decline in the average citation count per publication was evident during the years spanning 1987 to 2005, despite the concurrent increase in the number of publications. Subsequently, a discernible decrease in the average citation count per publication was observed post-2005.

### 3.2. Web of Science Core Collection Research Areas

The classification of papers into research areas within the Web of Science Core Collection (WoSCC) by Clarivate Analytics serves the purpose of simplifying the understanding of research topics. Each paper is assigned to at least one research field, and it is evident that the domains encapsulating primary themes in the study of floral scents have expanded over time. According to Web of Science data, the number of research fields within FS research has burgeoned from 5 in 1987 to an impressive 45 in 2022. Among these research areas, the top 10 in terms of productivity were identified as follows: Food Science Technology, Plant Sciences, Chemistry, Agriculture, Biochemistry, Molecular Biology, Environmental Sciences, Ecology, Science Technology, Other Topics, Pharmacology, Pharmacy, Entomology, and Nutrition Dietetics. These 10 research fields collectively accounted for 4589 out of the total 5049 publications, constituting an impressive 91% of the overall research output.

**Figure 2:**
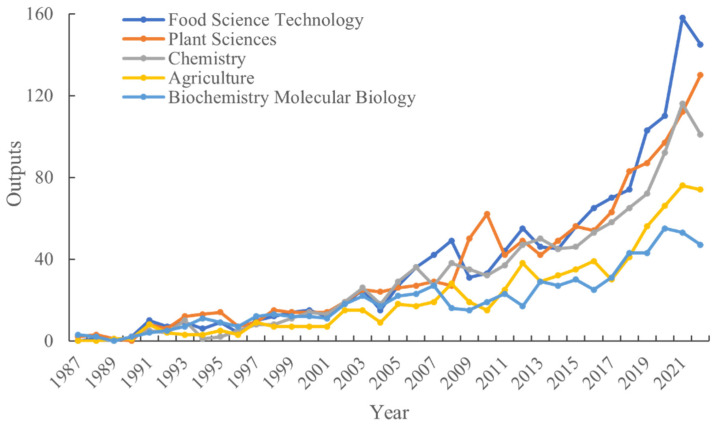
Temporal changes in the publication of the top five research areas in FS research. The classification of research areas is based on the WoSCC.

Figure 2 visually presents the annual progress of the top five most prolific research fields in the domain of FS research. Notably, all five of these areas exhibited exponential growth, echoing trends observed prior to 2005. Plant Sciences emerged as the most active research field, marked by the highest output increase. Post-2005, notable growth was observed in Food Science Technology, Plant Sciences, and Chemistry, with Food Science Technology experiencing particularly robust expansion. In contrast, the domains of Agriculture, Biochemistry, and Molecular Biology have demonstrated consistent growth, albeit at a relatively slower pace compared to the aforementioned areas.

### 3.3. Mapping the FS Topic Areas and Trends Using VOSviewer

**Figure 3:**
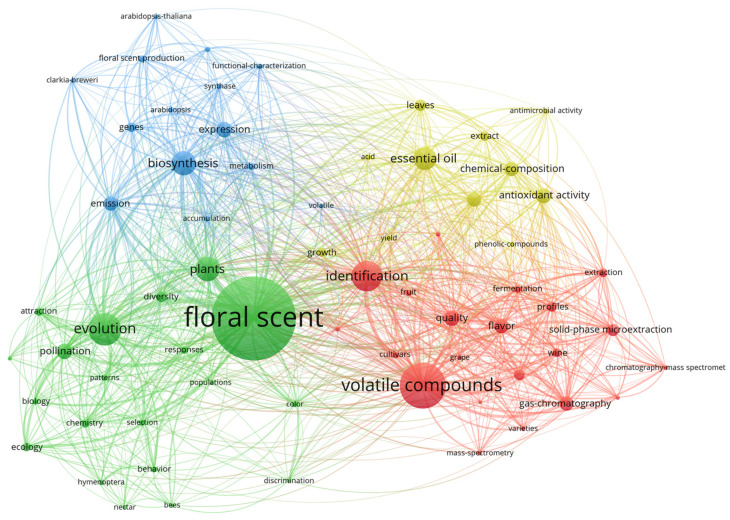
The co-occurrence network map of keywords in the worldwide scientific literature on FS. The size of each keyword (node) in the network is directly correlated with the frequency of its occurrences in the analysis of the literature. Colors are used to indicate clusters, in which keywords are grouped based on their interrelatedness in the network.

The exploration of FS topic areas and trends through the utilization of VOSviewer resulted in the categorization of the 66 extracted topic keywords into four distinct clusters. These clusters are sized proportionally to the frequency of keyword occurrences, as visually represented in Figure 3 and detailed in Table 1.

- Cluster 1 spans a diverse spectrum of topics, primarily centered on volatile compounds and their identification. Within this cluster, keywords encompass chemical attributes recognized through identification technologies such as “gas-chromatography”, “solid-phase microextraction”, “mass spectrometry”, and “chromatography-mass spectrometry”. Additionally, the cluster delves into qualities associated with identification, including “flavor”, “impact odorants”, and “olfactometry”, among others.
- Cluster 2, anchored by the central keyword “FS”, gravitates towards subjects emphasizing the integral role of pollinators in reproduction and evolutionary processes. This thematic cluster includes keywords like “Hymenoptera”, “populations”, “bees”, “pollination”, “attraction”, “selection”, “ecology”, and “discrimination”, among others, underscoring the ecological and evolutionary dimensions of FS research.
- Cluster 3 is dedicated to keywords associated with the synthetic mechanisms of FS, encompassing terms like “biosynthesis”, “expression”, “metabolism”, “synthase”, “accumulation”, and “functional-characterization”, among others. This cluster delves into the intricate processes underpinning the creation and regulation of floral scents.
- Cluster 4 encompasses keywords such as “essential oil”, “antioxidant activity”, and “antimicrobial activity”. These keywords are closely interconnected and signify the relevance of floral scents in applications related to essential oils and their various biological activities.

A discernible overlap between Cluster 1 (in red) and Cluster 2 (in green) is noticeable in Figure 3, with specific keywords bridging the two clusters within the network. Notably, this overlap contributes to an expansion of the overall network map. In contrast, Cluster 2 (in blue) and Cluster 3 (in yellow), situated at the upper section of the network graph, display limited intersection with Cluster 3 and Cluster 4, occupying a relatively smaller area within the map. The shared region between Cluster 1 and Cluster 2 underscores the interconnectedness of “volatile compounds” and “floral scent” in the research landscape.

**Figure 4:**
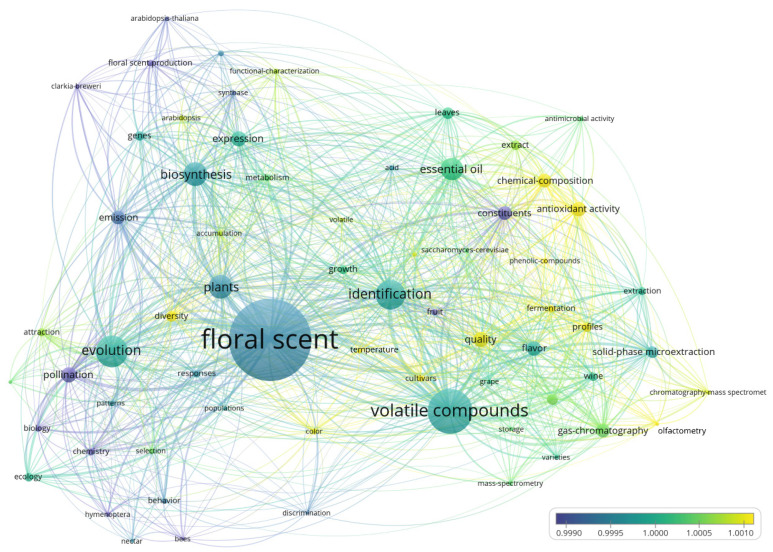
Temporal distribution of keywords in the co-occurrence network map. Visualizing the average publication year gradient from purple (older publications) to blue (publications equally distributed across the timespan 1987–2022) to yellow (more recent publications).

An overlay visualization map that incorporates the publication year of documents, introducing a temporal dimension to the interpretation of the co-occurrence network map of keywords, is presented in Figure 4. By placing keywords on a timeline, this approach allows for a comprehensive understanding of the evolution and progression of scientific research within the field of *FS*. Node colors correspond to the average publication year of each keyword, facilitating the identification of the most recent topics and research directions in FS. Over the course of 36 years, there has been a notable shift in keywords. Initially, the landscape was dominated by terms such as “pollination”, “biology”, “flavor”, “constituents”, and “emission”, among others. However, as time progressed, these keywords gave way to newer themes, including “diversity”, “color”, “temperature”, “cultivars”, “quality”, “fermentation”, “profiles”, “yield”, “olfactometry”, “functional-characterization”, “chemical-composition”, “phenolic-compounds”, and “antioxidant activity”. This transition reflects the dynamic nature of FS research and the evolving areas of focus within the scientific community.

### 3.4. Analysis of Country Network Cooperation

**Figure 5:**
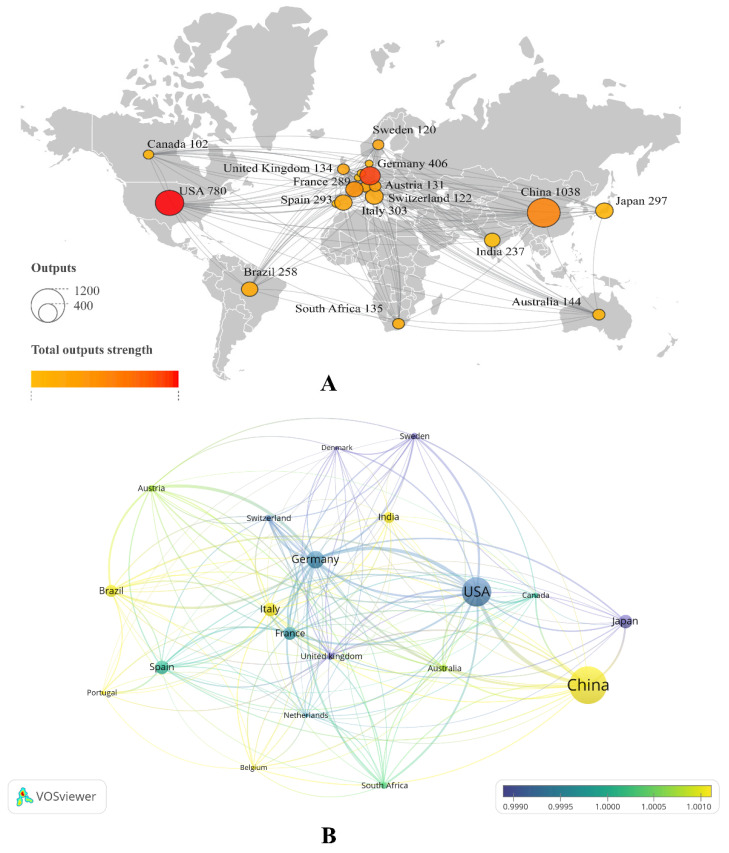
Analysis of top 20 countries’ cooperation network based on total link strength. Visualizing the total outputs strength from yellow to red in the map (**A**); visualizing the average publication year gradient from purple (older publications) to blue (publications equally distributed across the timespan 1987–2022) to yellow (more recent publications) (**B**).

The analysis of country network cooperation focused on the top 20 countries with the highest total link strength, as depicted in Figure 5A. This cooperation network diagram reveals that the current volume of articles is primarily concentrated in countries such as China, the United States, Germany, Japan, and Italy, among others. The most robust intensity of cooperation is observed among the USA, Germany, and China, which occupy the top three positions. Additionally, the research focus within this network is notably concentrated in European countries, including France, Spain, and Argentina, among others. Figure 5B provides an overlay visualization map that incorporates the publication year of documents, adding a temporal dimension to the interpretation of the co-author network map of countries. This analysis demonstrates a shift in prominence among countries over time. While Japan, the USA, and Germany were prominently featured in the past, there has been a notable transition towards countries such as China, Italy, India, and Brazil, among others, reflecting evolving patterns of international collaboration in research.

### 3.5. Dominant Institutions

**Figure 6:**
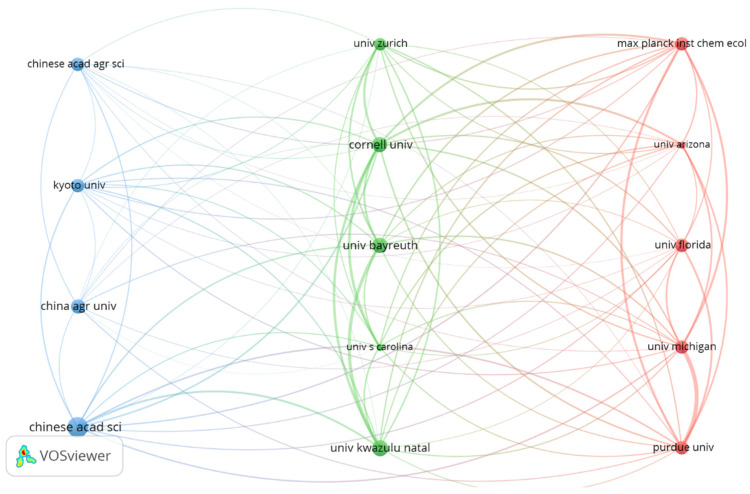
The network map showing the collaborations between 14 organizations in the top 10 in outputs and TLCS in the FS field.

This study has revealed that a staggering 4297 institutions worldwide have researched FS. The top 10 institutions made a collective contribution of 676 papers (including collaborative publications involving each institution). However, only four institutions surpassed the group average (67.6) outputs (Table 2). This indicates that the productivity of the top 10 institutions was distinctly uneven. Notably, the Chinese Academy of Sciences (CAS) emerged as the most prolific institution, publishing an impressive 122 articles during the study period. The University of KwaZulu-Natal (UKZN) and Cornell University (CU) came in second and third place with 78 and 79, respectively. Six institutions that made it to the top ten high-citation institutions list were also among the top ten in outputs (UM, PU, UB, MPICE, CU, and UKZN). All institutions from China were not ranked in the TLCS list. Most institutions in TLCS lists are from the USA and Germany. The top 10 organizations in outputs or in TLCS included 14 organizations, and a network diagram of cooperation among these organizations was made (Figure 6). The results showed that these institutions work very closely together. The top 10 authors in terms of publications and citations were screened (Table 3). Most authors ranked in outputs and TLCS were from the USA and Germany. R.A. Raguso, E. Pichersky, and S. Dotterl are among the authors with the relatively high number of publications and citations. 

## 4. Discussion

### 4.1. Technological Changes Drive FS Research Development

Technological advancements have played a pivotal role in driving the development of Fragrance Science (FS) research. Figure 1 illustrates the remarkable growth in FS research from 1987 to 2022, particularly after 2005. This surge can be attributed to various factors, with technology standing out as a key enabler in the research, production, application, and commercialization of fragrances. Until the 1990s, gas chromatography–mass spectrometry (GC–MS) instruments, vital for FS research, were prohibitively expensive for many research teams. However, the landscape changed with the advent of user-friendly software and the incorporation of artificial intelligence, coupled with the mass production of GC–MS machines, which led to reduced costs and simplified operation [38]. This innovation empowered plant biologists to distinguish floral volatiles from a multitude of other plant species. An array of techniques, such as GC-Olfactometry (GC-O), high-performance liquid chromatography (HPLC), liquid chromatography–mass spectrometry (LC–MS), nuclear magnetic resonance (NMR), and more, were developed for detecting volatile compounds in FSs [39,40,41,42]. Supercritical carbon dioxide (SC-CO2) [43], ultrasound-assisted maceration (UM) [44], and direct thermal desorption–gas chromatography–mass spectrometry (DTD–GC–MS) [45] are very popular extraction and analytical methods for flavors. Scientists harnessed these analytical methods to identify and analyze the aroma components of various flowers, shedding light on their chemical composition and structure, aiding in the mimicry and synthesis of these aromas. The application of electronic noses and electronic tongues, as sensory technologies, allowed for the detection and evaluation of fragrance characteristics [46,47]. These technologies facilitated the assessment of aroma quality and product standards. Recent years have witnessed significant progress in FS research, thanks to molecular biology technologies like genomics, metabolomics, proteomics, and transgene editing technologies. These innovations, combined with the human impetus, such as the establishment of the FS panel at the 2002 Gordon Research Conference [48], fostered new collaborations, inspiring plant biochemists and physiologists to delve deeper into FS biochemistry studies. This, in turn, led to practical applications of FS research within the field of ecology, completing a full circle of scientific advancement.

As the production of FS research outputs soared, the trend in article citations followed an inverse trajectory (Figure 1). This phenomenon could be attributed to the increased annual outputs providing scientists with a wider pool of references to choose from. Additionally, the time difference in citations played a role, with more recent research outputs having lower citation numbers, despite their significant impact. Older research outputs became less relevant as new ones emerged. The average citation-per-publication counts fluctuated based on the annual number of publications and their quality within the field [49].

### 4.2. Research Focuses of FS

In the realm of FS research, keywords were categorized into four distinct groups, i.e., volatile components, floral scent, synthesis, and essential oils (Figure 3), indicating the interconnectedness of these aspects in research content and topics. Notably, the presence of the keywords “plants”, “evolution”, and “biology” suggests a focus on understanding the relationship between floral characteristics and the biological evolution of plants. Researchers have explored how factors like size, color, and organ structure of flowers evolve in response to environmental changes [50,51,52]. Furthermore, the study conducted by Adler et al. suggests that FS undergoes stronger selective pressures than traditionally measured floral morphological traits [53]. For instance, specific FSs attract particular pollinators, with larger bees being drawn to certain butterfly bush species, underscoring the vital role of FSs in pollinator attraction [54]. FSs are adept at attracting pollinators rapidly, especially those that are nocturnal or have to travel long distances. For instance, the nocturnally active sky moth pollinator *Manduca sexta* relies on olfactory cues provided by Petunia axillaris, while nocturnal bees use olfactory cues to locate *Campomanesia phaea* [55,56]. 

Keywords such as “behavior”, “chemistry”, “patterns”, and “reproductive isolation” linked with “floral scent” hint at researchers’ growing interest in understanding the connections between pollinator behavior, floral scent chemistry, and their roles in reproductive isolation mechanisms. Floral chemistry helps plants improve their chances of pollination success by influencing pollinator perception and behavior. The spatial chemical signatures of Silene latifolia floral scents guide pollinators to the location and distance of flowers, helping them to locate flowers more effectively [57]. *Ceropegia sandersonii* flowers mimic the release of bee alarm substances to lure food-stealing fly pollinators [58]. Most *morphophallus species*, Araceae and Orchidaceae, attract pollinators by releasing sexually deceptive signals [59]. Over long periods of evolution, plants have gradually adapted to specific pollination methods and pollinators by releasing specific compounds [60]. For example, insect-pollinated flowers release a wider variety of terpenes and benzene compounds compared to wind-pollinated flowers [61]. Beta-stilbene and beta-element are attractive to Asian wasps but not to bumblebee and hornet species [62]. *Philodendron fragrantissimum (Hook.) G. Don* attract *Cyclocephala simulatrix Höhne* for specialized pollination by releasing methyl benzoate, (Z)-jasmonone, and dehydrojasmonone [63]. Plants can adapt the timing, space, amount, and type of FSs released to the sensory characteristics of pollinators [57,64,65] and the quality and quantity of rewards (e.g., nectar, pollen, etc.) [66,67] offered to maximize attraction and retention of specific pollinators [68]. Bats, bees, butterflies, hummingbirds, and moths are known to be able to perceive the scent of flowers and distinguish between odor differences [69,70,71,72]. These explain, for example, that flowers with moth pollination syndrome exhibit high rates of odor emission in the late afternoon [73]. *Gloxinia perennis* adapts to Eulaema bees through floral chemistry and daily fluctuations [74] et al. The optimization of behavioral interactions helps to improve the reciprocal relationship between plants and pollinators, thus increasing the reproductive success of plants. There may also be convergent evolution of FSs and pollination vectors such as Apidae and Euglossini [75]. Differences in floral chemical characteristics may result in failure to attract the same pollinators and ensure transfer of the same pollen [76], leading to reproductive isolation, which has been demonstrated in many plants [77,78,79]. FSs act as phytochemical signals that attract pollinators, vector natural enemies, or phytophagous insects and create complex networks of interactions between plants or between plants and other organisms [80].

The convergence of “volatile compounds”, “biosynthesis”, and “essential oil” with “floral scent” underscores researchers’ dedication to unraveling the formation, composition, and functions of FSs [81,82]. Through in-depth exploration of biosynthetic pathways and mechanisms related to plant volatile compounds and the analysis of essential oil components [83], scientists are gaining insights into the pivotal role of FSs in plant biology and ecosystems, thereby advancing the fields of plant ecology, biochemistry, and biotechnology. These findings not only hold biological significance but also offer practical applications in plant protection and ecosystem maintenance [84,85]. 

### 4.3. Trends in FS Research

Over a span of 36 years, the landscape of FS research has evolved significantly, as evident from the shift in prominent keywords. Initially, terms like “pollination”, “biology”, “flavor”, “constituents”, and “emission”, among others, dominated the field. However, more recently, the focus has shifted towards keywords such as “diversity”, “color”, “temperature”, “cultivars”, “quality”, “fermentation”, “profiles”, “yield”, “olfactometry”, “functional-characterization”, “chemical composition”, “phenolic compounds”, and “antioxidant activity”. These newer keywords, highlighted in yellow, represent the current hot areas in evolutionary adaptation, chemical identification, biosynthetic processes, and essential oils fields (Figure 4).

The transition from keywords such as “pollination”, “pattern”, and “attraction” to “diversity” signifies a broadening of researchers’ perspectives within the realm of FS research. The focus has expanded beyond the pollination process to encompass a broader spectrum of ecosystem interactions. Researchers now investigate the evolutionary adaptations of plants, species diversity, and ecosystem stability. Floral biology has emerged as a multidisciplinary field, drawing expertise from various domains [1]. Collaboration and interaction with ecologists, chemists, biodiversity specialists, and scholars from diverse fields have become integral signs of the expanding horizons of FS research [5,86]. Ecologists leverage floral studies to assess how plants adapt to climate change and environmental stress, providing valuable scientific evidence and recommendations to address ecological challenges posed by global climate change [87], such as factors like sesquiterpene emission being influenced by ambient temperature [8]. Abbas et al. suggested that the increase in volatile organic compound emissions with temperature may be related to future heat tolerance adaptation in plants [82]. Climate change may affect the sensory ecology and behavior of inserts [88]. Researchers have investigated the impact of pollination vectors (such as bees and flies) on the ecology and evolution of flowers [1,86]. Typical species isolation of plant taxa with orchids is based on adaptations to different pollinators mediated by FSs [89]. The molecular basis of signaling differences in the evolution of floral isolation is important for its emergence and potential fixation between populations (or species) [68], and differences based on simple mutations are expected to occur more frequently, leading to higher fitness, as demonstrated in the evergreen azalea [90]. FSs are complex multifunctional signals that pollinators often use in combination with other signals (e.g., color) [91]. Researchers are increasingly inclined to intertwine FS with floral phenotypic traits, to analyze how pollination syndromes dictate pollinator preferences [92]. There is also a growing curiosity about the mechanisms that might exert negative influences on ecosystem stability and diversity—factors ranging from environmental change to habitat degradation and the precarious decline of pollinator populations, which pose a significant threat to these vital contributors to our ecosystems [93,94].

In addition to shifts in FS research trends, related topics have also seen significant changes in research focus. Terms like “profiles”, “yield”, “quality”, and “fermentation” have become hot keywords in the context of “volatile compounds” related to “floral scent”. This shift reflects an expanded interest from purely sensory properties of floral scent components to broader aspects, especially in production and processing. Researchers are increasingly concerned with optimizing the overall quality and efficiency of food fermentation processes [95]. This includes areas such as selecting the right yeast strains and employing fermentation techniques to enhance the aroma quality and diversity of food and beverages, aligning with market demands and improving economic efficiency. For example, Zhang et al. increased the concentration of phenolic acids in Petit Manseng wines via co-fermentation with *Saccharomyces cerevisiae* using *Torulaspora delbrueckii* and *Hanseniaspora vineae* to improve the aromatic profile and safety quality [96]. The high publication count of FS research in Food Science and Technology underscores the wide range of applications of floral aroma in the food industry [97,98].

Within the realm of floral volatiles, there are three main groups, including terpenoids, phenolic compounds, and aliphatic derivatives [99]. Terpenoids are a large group of naturally volatile organic compounds usually found in distilled plants extracts. They play various important physiological and ecological roles in plants, mainly by luring natural enemies against plant pathogens and herbivores, etc. [100]. Xu et al. demonstrated that volatile sesquiterpenes and monoterpenes in *C. morifolium* act as chemical signals primarily responsible for indirect defense, attracting predatory and parasitic natural enemies of herbivores [101]. Terpenoids are structurally diverse, including monoterpenes, sesquiterpenes, triterpenes, etc. Their chemical structure and volatile properties give them unique flavors and pharmacological activities [102]. The prominence of keywords “phenolic compounds” and “antioxidant activity” within the context of “essential oil” associated with “floral scent” indicates a specific research interest in exploring the impact of compounds within essential oils on combatting oxidative stress and cellular damage. These properties hold potential for various applications, including in medicine, nutraceuticals, and cosmetics [103,104,105]. It is important to note that some volatile compounds may have toxic effects on humans or other organisms, and attention must be paid to the health and safety hazards posed by volatile compounds during their use. For example, estragole has been associated with the development of malignant tumors in rodents [106]. One of the main constituents of essential oils, the sesquiterpenoid ledol, may have a stimulating effect similar to caffeine in low concentrations in drinks, but in high doses, it affects the central nervous system, leading first to psychomotor stimulation, then to epilepsy and convulsions, and finally to paralysis, respiratory problems, and even death [107,108]. Therefore, caution needs to be exercised and relevant safety guidelines followed when using these compounds. Kumar et al. have provided us with a database of medicinal and aromatic plant aroma molecules with phytochemical and therapeutic potential (http://bioinfo.cimap.res.in/aromadb/) [109], which contains properties related to aroma molecules, hazard identification, exposure control and personal protection, physical and chemical properties, and toxicological and ecological information. Additionally, allergic reactions need to be considered, especially odor allergens, as 26 volatile ingredients are included in the list of potentially allergenic substances according to the EU Cosmetics Regulation [110], such as benzyl alcohol, cinnamaldehyde, citronellol, eugenol, linalool, and limonene are commonly detected allergens [111].

In recent years, the development of biotechnology has revealed more functional features of odor genes in biological evolution, compound synthesis, etc. For example, Wang et al. revealed the major force played by tandem duplication in the evolutionary and adaptive mechanisms of *Rhododendron* by using PacBio sequencing and Hi-C technology to functionally characterize the *R. ovatum* genome [90]. Amrad et al. showed that the genetic basis for variation in the trait of floral odor emission in *Petunia* spp. (Solanaceae) is mainly related to cinnamate-CoA ligase (CNL) [112]. Using virus-induced gene silencing (VIGS), Shi et al. demonstrated that long non-coding RNAs (lncRNAs) play an important role in the production of rose scent [113]. The integration of molecular biology, genomics, ecosystem simulation, and biotechnology tools is helping to understand the mechanisms and effects of floral scents and ecosystem responses [114,115]. These efforts contribute to accelerate plant molecular breeding and metabolic engineering by uncovering the molecular processes behind FS production and emission, ultimately improving FS production and essential oils extraction from plants [116,117]. The expected outcome is an enhanced attractiveness to pollinators, which, in turn, is expected to improve agronomic crop yields [118,119]. Interdisciplinary cooperation and international collaboration have significantly propelled the field of floriculture. This study reveals that China, the USA, and Germany lead in publications in this field, with other countries like Italy and India also making significant contributions. Most institutions and authors from the USA and Germany are top 10 in terms of outputs and TLCS. It is worth noting that since the research in this paper mainly focuses on floral scents, the keyword search tends to miss other researchers with outstanding contributions, such as some famous essential oil researchers K. Hüsnü Can Başer (Turkey), Luigi Mondello (Italy), Joshinori Asakawa (Japan), Robert P. Adams (USA), Gerhard Buchbauer (Austria), etc. And many researchers working on FS primarily study whole plant volatiles in the context of herbivory, plant–plant communication, or tri-trophic interactions, such as Ian T. Baldwin (Germany), Andre and Danny Kessler (USA, Germany), and Marcel Dicke (Netherlands). A total of 14 organizations in top 10 output or TLCS work very close. This may indicate that research in the FS field is concentrated in a small number of organizations or authors with a high number of publications and influence. For other countries, such as China, they have had a high number of publications in recent years but lack influence. Some other countries are also paying more attention to FS research, such as Italy, Brazil, India, etc. Strengthening cooperation among nations holds substantial implications for enhancing the diversity and stability of ecosystems within the context of interdisciplinary collaboration.

## 5. Conclusions

**Figure 7:**
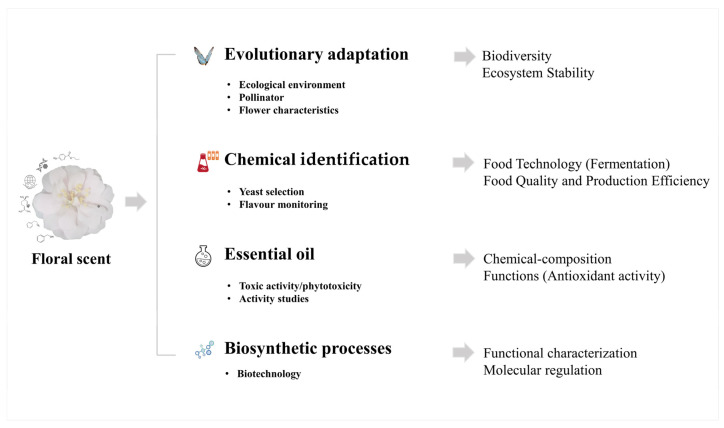
The potential future research trends and areas of FS based on the published articles.

The rapid evolution of FS research is undeniably indebted to technological advancements driving the field to unprecedented heights. However, while the surge in published articles is impressive, it is equally vital to maintain a steadfast commitment to the quality of these contributions. By the year 2022, FS research firmly gravitated towards four pivotal domains: the evolutionary facets, chemical compositions, biosynthetic processes, and essential oils. Figure 7, a visual representation of our comprehensive analysis, vividly illustrates the potential framework for the future of FS research based on published articles. The research trend is to investigate the evolutionary adaptive mechanism of plants in the context of ecological environment, pollination medium, flower characteristics, etc.; to optimize yeast selection so as to improve product quality and productivity; to focus on the antioxidant activity of phenolic compounds and the safety mechanism of the related components; as well as the use of biotechnological techniques to characterize the function of the related genes or molecular regulation. The cumulative effect of these endeavors, coupled with the continuous refinement of research methodologies and biotechniques, and bolstered by robust international interdisciplinary collaborations, promises a more profound understanding of FS’s multifaceted roles. This understanding extends to its pivotal contributions in revealing genetic basis, promoting biodiversity conservation, enhancing ecosystem stability and diversity, driving agricultural productivity, securing the sustainability of the food supply chain, and catalyzing advancements in biotechnology and drug discovery.

The article creates a holistic overview database for those who are new to the subject of floral scent (https://www.webofscience.com/wos/woscc/summary/65b2c9b4-a66d-40cf-b290-e6981a2d9d30-7213ff82/relevance/1, accessed on 5 April 2023) and provides a referable network of countries and author collaborations. As the first bibliometric study of FS research, this analysis is exploratory, but sets the stage for the future research landscape. Despite limitations (bibliometric mapping constraints), map analysis represents a valid tool that can support experts in various countries as they strive to improve their knowledge and pursue novel projects in the field of FS research.

## Figures and Tables

**Table 1:** Clusters and relative keywords resulting from co-occurrence analysis.

Cluster	Color	Keywords	Occurrences	Links	Total Links
1	Red	volatile compounds	770	65	1942
		identification	484	65	1247
		quality	230	54	530
		flavor	217	56	574
		gas chromatography	207	51	629
		solid-phase microextraction	178	46	490
		impact odorants	162	43	425
		profiles	136	48	377
		wine	131	41	366
		extraction	122	40	345
		fermentation	102	42	260
		fruit	98	54	239
		cultivars	93	49	263
		temperature	69	52	184
		mass spectrometry	68	38	167
		varieties	68	40	183
		saccharomyces cerevisiae	67	38	162
		chromatography–mass spectrometry	65	26	191
		grape	65	41	177
		olfactometry	52	32	180
		storage	51	36	130
2	Green	floral scent	1497	65	3459
		evolution	522	60	1375
		plants	381	62	879
		pollination	230	51	583
		diversity	159	59	400
		ecology	128	32	341
		attraction	119	40	304
		chemistry	111	40	294
		responses	101	47	226
		behavior	87	34	181
		biology	87	33	223
		color	85	51	240
		selection	82	37	220
		patterns	81	40	211
		hymenoptera	73	29	161
		populations	57	43	145
		bees	56	27	129
		discrimination	56	36	133
		reproductive isolation	54	22	105
		nectar	53	27	113
3	Blue	biosynthesis	380	60	1092
		expression	229	50	604
		emission	211	56	700
		genes	136	44	369
		floral scent production	95	32	200
		metabolism	92	50	269
		gene expression	78	36	171
		accumulation	74	48	210
		functional characterization	74	40	218
		arabidopsis	68	30	170
		synthase	67	38	218
		arabidopsis thaliana	64	26	140
		clarkia breweri	60	27	185
		volatile	53	47	146
4	Yellow	essential oil	358	60	791
		antioxidant activity	220	47	477
		constituents	214	54	500
		chemical composition	213	49	455
		leaves	172	53	403
		extract	142	38	255
		growth	131	53	230
		acid	76	47	164
		antimicrobial activity	73	29	137
		phenolic compounds	69	40	156
		yield	63	36	119

**Table 2:** The top 10 institutions with the highest productivity and citation impact.

Institution	Country	Outputs	Institution	Country	TLCS
CAS	China	122	UM	USA	1579
UKZN	South Africa	78	PU	USA	1481
CU	USA	79	UB	Germany	1194
UB	Germany	69	MPICE	Germany	851
CAU	China	59	CU	USA	727
KU	Japan	55	UKZN	South Africa	637
UM	USA	55	USC	USA	629
CAAS	China	54	UA	USA	576
MPICE	Germany	53	UF	USA	443
PU	USA	52	UZ	Switzerland	415
Average		67.6	Average		853.2

Note: CAS: Chinese Academy of Sciences, China; UKZN: University of KwaZulu-Natal, South Africa; CU: Cornell University, USA; UB: University Bayreuth, Germany; CAU: China Agricultural University, China; KU: Kyoto University, Japan; UM: University of Michigan, USA; CAAS: Chinese Academy of Agricultural Sciences, China; MPICE: Max Planck Institute for Chemical Ecology, Germany; PU: Purdue University, USA; USC: University of South Carolina, USA; UA: University of Arizona, USA; UF: University of Florida, USA; and UZ: University of Zurich, Switzerland.

**Table 3:** The top 10 authors with the highest productivity and the top 10 authors with the highest TLCSx (Total Local Citation Score excluding self-citations).

Author	Institution	Outputs	Author	Institution	TLCSx
S. Dotterl	UB, Germany	116	N. Dudareva	PU, USA	1346
R.A. Raguso	USC, USA	79	R.A. Raguso	USC, USA	1304
S.D. Johnson	UKZN, South Africa	64	E. Pichersky	UM, USA	1216
E. Pichersky	UM, USA	53	S. Dotterl	UB, Germany	700
F.P. Schiestl	UZ, Switzerland	51	C.M. Kish	PU, USA	626
N. Dudareva	PU, USA	49	A. Jurgens	UB, Germany	602
F. Chen	UM, USA	48	F.P. Schiestl	UZ, Switzerland	580
A. Jurgens	UB, Germany	43	J.T. Knudsen	UG, Sweden	567
N. Watanabe	US, Japan	37	L. Tollsten	UG, Sweden	414
J. Wang	UM, USA	34	E. Lewinsohn	UM, USA	362

Note: UB: University Bayreuth, Germany; USC: University of South Carolina, USA; UKZN: University of KwaZulu-Natal, South Africa; UM: University of Michigan, USA; UZ: University of Zurich, Switzerland; PU: Purdue University, USA; US: University of Shizuoka, Japan; and UG: University of Göteborg, Sweden.

## Data Availability

Data are contained within the article.
